# Supplementary material for: Quantification of signal amplification for receptors: the K d/EC50 ratio of full agonists as a gain parameter
Source: Front Pharmacol. 2025 Apr 8;16:1541872. doi: 10.3389/fphar.2025.1541872 (PMC12011844; doi:10.3389/fphar.2025.1541872)

SUPPLEMENTARY INFORMATION

Quantification of Signal Amplification for Receptors: The K_d/EC_{50} Ratio of Full Agonists as a Gain Parameter

Peter Buchwald

Department of Molecular and Cellular Pharmacology and Diabetes Research Institute,
Miller School of Medicine, University of Miami, Miami, FL, USA

- Appendix 1. Derivation of the horizontal shift between two sigmoid functions.

Appendices

Appendix 1. Horizontal shift between two sigmoid functions.

Assuming straightforward hyperbolic functions (sigmoidal on the semi-log scale) both for the occupancy and the response, the corresponding fractional occupancies are:

$$f_{\text{occup}} = e_{100}\frac{[L]}{[L]+K_{\text{d}}} = e_{100}\frac{\frac{[L]}{K_{\text{d}}}}{\frac{[L]}{K_{\text{d}}}+1} \tag{A1}$$

$$f_{\text{resp}} = e_{\max}\frac{[L]}{[L]+K_{\text{obs}}} = e_{\max}\frac{\frac{[L]}{K_{\text{obs}}}}{\frac{[L]}{K_{\text{obs}}}+1} \tag{A2}$$

Here, e_{max} and e_{100} are the corresponding maxima for response and occupancy, respectively (with the assumption that occupancy always reaches its full maximum, hence the notation of e_{100} for 100%), and K_{obs} and K_d the half-maximal concentrations. To obtain the ligand concentration values that produce the same (fractional) response and occupancy $[L]_o$ and $[L]_r$, respectively, one can set $f_{occup} = f_{resp}$ (any value as long as they are the same):

$$f_{\text{occup}} = f_{\text{resp}} \;\Rightarrow\; e_{100}\frac{\frac{[L]_{\text{o}}}{K_{\text{d}}}}{\frac{[L]_{\text{o}}}{K_{\text{d}}}+1} = e_{\max}\frac{\frac{[L]_{\text{r}}}{K_{\text{obs}}}}{\frac{[L]_{\text{r}}}{K_{\text{obs}}}+1} \tag{A3}$$

For a full agonist, response also reaches its full maximum (just as occupancy does), $e_{max} = e_{100} = 1$ (100%), so that f_{occup} and f_{resp} follow the same functional form (eqs. A1 & A2) just with a shifted argument. Thus, from eq. A3:

$$full\ agonist\colon\ e_{\max} = e_{100} = 1 \;\Rightarrow\; \frac{[L]_{\text{o}}}{[L]_{\text{r}}} = \frac{K_{\text{d}}}{K_{\text{obs}}} = \kappa = g_K \tag{A4}$$

Thus, for a full agonist, the K_d/K_{obs} (i.e., K_d/EC_{50}) ratio corresponds to the g_K gain parameter as defined earlier, and on the typical semi-log plot, it corresponds to the horizontal shift between occupancy and response, which is the same along any horizontal line:

$$full\ agonist\colon\ \log g_K = \log K_{\text{d}} - \log K_{\text{obs}} \tag{A5}$$

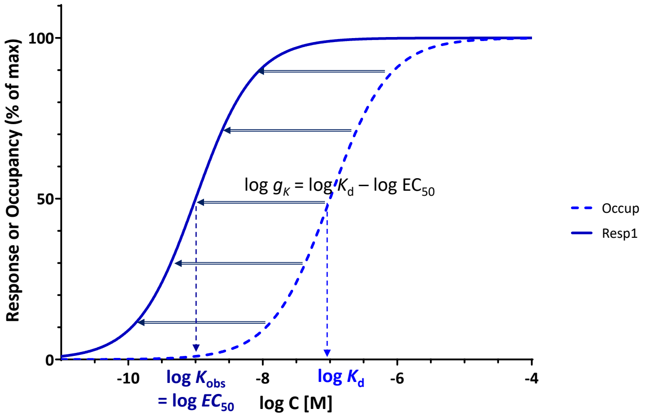

Supplement: Supplementary file 1 [file DataSheet1.pdf]
